# Maternal Stressors and Social Support in Early Pregnancy and the Risk of Stillbirth Among Fetuses With Birth Defects: Exploring Effect Modification by Race/Ethnicity

**DOI:** 10.1002/bdr2.70096

**Published:** 2026-07-25

**Authors:** Meredith M. Howley, Jada M. Scott, Eva M. Williford, Uma M. Reddy, Eleni A. Papadopoulos, Suzan L. Carmichael, Chris M. Cunniff, A. J. Agopian, Nahed Elhassan, Wendy N. Nembhard, Sarah C. Fisher, for the National Birth Defects Prevention Study and Birth Defects Study to Evaluate Pregnancy exposureS

**Affiliations:** ^1^ Birth Defects Registry New York State Department of Health Albany New York USA; ^2^ Department of Epidemiology and Biostatistics, College of Integrated Health Sciences University at Albany Albany New York USA; ^3^ Department of Obstetrics and Gynecology Columbia University Irving Medical Center New York New York USA; ^4^ Department of Pediatrics Stanford University School of Medicine Stanford California USA; ^5^ Department of Obstetrics and Gynecology Stanford University School of Medicine Stanford California USA; ^6^ Department of Pediatrics Weill Cornell Medical College New York New York USA; ^7^ Department of Epidemiology UTHealth School of Public Health Houston Texas USA; ^8^ Department of Pediatrics, Division of Neonatology University of Arkansas for Medical Sciences Little Rock Arkansas USA; ^9^ Department of Epidemiology, Fay W. Boozman College of Public Health University of Arkansas for Medical Sciences Little Rock Arkansas USA

## Abstract

**Background:**

Prenatal stress and social support are associated with adverse birth outcomes, including stillbirth, in the general population. Their relationship to stillbirth among fetuses with birth defects is unclear. Using data from the National Birth Defects Prevention Study and the Birth Defects Study To Evaluate Pregnancy exposureS, we examined associations between maternal stress and social support and the risk of stillbirth among fetuses with birth defects and whether these associations differed by maternal race/ethnicity.

**Methods:**

Participants self‐reported exposure to stressors and social supports experienced 3 months before conception through the first trimester. Using log‐binomial models, we estimated relative risks (RR) and 95% confidence intervals (CI) of stillbirth associated with high stress and low social support, adjusting for maternal age and race/ethnicity. We assessed additive and multiplicative effect measure modification by maternal race/ethnicity.

**Results:**

Neither stress nor social support was associated with stillbirth among fetuses with birth defects. Although we did not observe interaction, we observed increased risk of stillbirth among non‐Hispanic Black women with low stress (RR = 2.1, 95% CI = 1.3–3.6), Hispanic women with low stress (1.4, 1.0–2.1), and non‐Hispanic Black women with high stress (2.8, 1.4–5.2) compared to non‐Hispanic White women with low stress. We observed a similar pattern of results for low social support.

**Conclusions:**

Stress and social support were not associated with stillbirth in fetuses with birth defects. We observed elevated risk of stillbirth among non‐Hispanic Black women regardless of stress and social support levels.

## 1 Introduction

Approximately 20,000 stillbirths (fetal deaths occurring at ≥ 20 weeks' gestation) occur in the United States (US) each year (Gregory et al. 2024). Fetuses with birth defects have an increased risk for stillbirth compared to the general population and account for approximately 14% of all stillbirths (Frey et al. 2014; Heinke et al. 2020; Stillbirth Collaborative Research Network Writing Group 2011a; Silver and Reddy 2024). Yet, little is known about other stillbirth risk factors among pregnancies with a major birth defect (Frey et al. 2014; Heinke et al. 2020; Kerr et al. 2022). Given that many birth defects are identified prenatally, a better understanding of factors that impact the risk of stillbirth among fetuses with a birth defect may offer opportunities to improve pregnancy outcomes (Kerr et al. 2022).

Among the general population, several risk factors for stillbirths have been identified (Gregory et al. 2024; Reddy et al. 2010; Stillbirth Collaborative Research Network Writing Group 2011b), which may provide insight into potential associations to investigate among fetuses with birth defects. The association between maternal race/ethnicity and stillbirth is well‐established, with non‐Hispanic Black women experiencing two times the stillbirth risk of non‐Hispanic White women (Pruitt et al. 2020). Psychosocial stress has also been associated with an increased risk of stillbirth in the general population, as well as other adverse pregnancy outcomes (Wisborg et al. 2008; Hogue et al. 2013; Carmichael et al. 2007, 2014, 2017; Weber et al. 2020; Dolk et al. 2020; Li et al. 2017; Hogue 2016). A Stillbirth Collaborative Research Network study observed that women who reported experiencing the most stress in pregnancy had over two times the odds of a stillbirth compared to those who reported no stress (Hogue et al. 2013). Additionally, there is evidence that the association between stress and stillbirth may be modified by maternal race/ethnicity (Hogue et al. 2013). Social support may mitigate the negative impacts of stress, as multiple studies have shown that greater emotional support is associated with lower levels of perceived stress, anxiety, and depression during pregnancy (Wang et al. 2023; Al‐Mutawtah et al. 2023). Additionally, several studies also suggest that the link between high stress and adverse birth outcomes is attenuated among those who report higher social support compared to those who reported lower social support (McDonald et al. 2014; Suarez et al. 2003; Carmichael et al. 2003). Yet, findings are mixed, as others have not observed that social support offsets the negative impacts of stress during pregnancy (Carmichael et al. 2014; Weber et al. 2020).

While maternal stress and social support may impact the risk of stillbirth, their role as a risk factor among fetuses with major birth defects is not well understood. Pregnancies affected by birth defects represent a distinct clinical context with additional medical and psychosocial complexities that may influence stillbirth risk. Thus, we used data from two large population‐based studies to estimate the association of reported stress and social support with the occurrence of stillbirth among fetuses with birth defects. Given the need to better understand the observed racial disparities in stillbirth (Willinger et al. 2009), we also assessed whether the associations between stress/social support and stillbirth among fetuses with birth defects differed by maternal race/ethnicity on the additive and multiplicative scales.

## 2 Methods

We combined data from the National Birth Defects Prevention Study (NBDPS) and the Birth Defects Study to Evaluate Pregnancy exposureS (BD‐STEPS). Detailed methods can be found elsewhere (Reefhuis et al. 2015; Tinker et al. 2015). Briefly, the NBDPS includes data on pregnancies with a delivery on or after October 1, 1997, and with an estimated date of delivery (EDD) on or before December 31, 2011 in ten participating sites (Arkansas, California, Georgia, Iowa, Massachusetts, New Jersey, New York, North Carolina, Texas, and Utah). Our analyses included NBDPS participants with EDDs from January 2006 to December 2011, the years in which questions regarding stress and social support were asked. BD‐STEPS includes data on pregnancies with a delivery from January 1, 2014 through August 31, 2015 and July 1, 2016 through December 31, 2021 in seven sites (Arkansas, California, Georgia, Iowa, Massachusetts, New York, and North Carolina). In both studies, liveborn, stillborn or terminated pregnancies (at any gestational age) affected by an eligible major structural birth defect, excluding those attributed to a known chromosomal or single‐gene abnormality, were ascertained through birth defects surveillance programs (Reefhuis et al. 2015; Tinker et al. 2015). Live births without any major birth defects were randomly selected from hospital records (NBDPS) or birth certificates (NBDPS and BD‐STEPS) in the same catchment area as the cases. Between 6 weeks and 24 months after the EDD, eligible women were invited to complete a computer‐assisted telephone interview in English or Spanish to collect pregnancy exposure information. Each study site and the Centers for Disease Control and Prevention received institutional review board approval for both studies and obtained informed consent.

**FIGURE 1:**
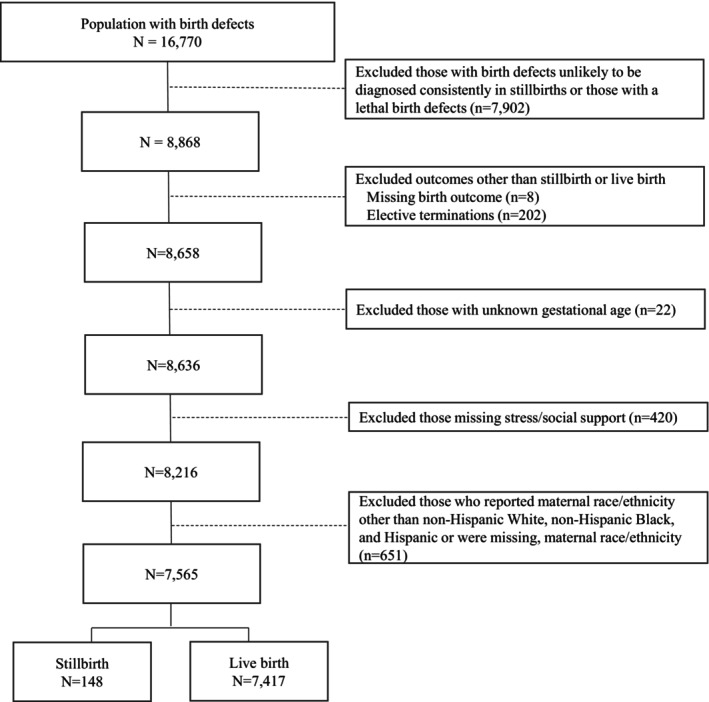
Study population, National Birth Defects Prevention Study (2006–2011) and Birth Defects Study To Evaluate Pregnancy exposureS (2014–2021).

We limited the analysis to fetuses with birth defects resulting in a live birth or stillbirth. Given previous work on stillbirth risk in NBDPS (Heinke et al. 2020), we excluded fetuses from our analysis with birth defects that are frequently diagnosed based only on postnatal findings or are otherwise unlikely to be diagnosed consistently in stillbirths, which included biliary atresia, small intestinal atresia, colonic or anorectal atresia, craniosynostosis, cerebellar hypoplasia, hypospadias, glaucoma, cataracts, choanal atresia, undefined limb deficiency, and all isolated heart defects (Figure 1). We also excluded fetuses with anencephaly, given it is a lethal anomaly (Centers for Disease Control and Prevention 2024).

In both studies, women were asked five questions about whether they experienced stressful life events and three questions about social supports during the 3 months before pregnancy through the third month of pregnancy (specific questions are listed in Table S1). These questions were taken from the Kaiser Permanente/California Department of Health Study of Pregnancy and Stress, and largely parallel many of the questions in existing, validated stressful life events assessment tools (Carmichael et al. 2007; Holmes and Rahe 1967; Sarason et al. 1978; Newton and Hunt 1984). Our analysis was restricted to NBDPS and BD‐STEPS participants who were not missing responses to these questions (Figure 1). In our main analysis, we dichotomized stress as low (0–1 reported stressors) versus high (2–5 reported stressors) and social support as low (0–2 supports) versus high (3 supports). We also examined each reported stressor and social support element individually (present vs. absent). Lastly, we examined the stress and social support variables in combination as a four‐level variable: high stress/low support, high stress/high support, low stress/low support, and low stress/high support (reference group).

The NBDPS race/ethnicity data collection methods were consistent with the National Center for Health Statistics' categorization (Ingram et al. 2003). Women were asked about their race/ethnic group and received an interviewer prompt that they were able to select more than one category: American Indian or Alaska Native; Asian; Black or African American; Hispanic or Latina; Native Hawaiian or Other Pacific Islander; and White. In BD‐STEPS, race and ethnicity were separate questions. Women were first asked if they were Hispanic or Latina and then asked to describe their race and received an interviewer prompt that they were able to select more than one category: American Indian or Alaska Native; Asian; Black or African American; Native Hawaiian or Other Pacific Islander; and White. Due to small numbers of stillbirths and livebirths among women of other race/ethnicity categories, our analysis was restricted to women who reported being non‐Hispanic White, non‐Hispanic Black, or Hispanic.

We compared stillbirths and livebirths with birth defects across selected maternal characteristics. We considered any absolute differences of > 5% between groups to be notable. We used log‐binomial models to estimate crude and adjusted relative risks (RR) and 95% confidence intervals (CI) for the association between both exposures and the risk of stillbirth. We ran three models: one for high stress, one for low social support, and one for the four‐level combined stress and social support exposure variable. We determined potential covariates *a priori* from the literature and evaluated potential confounders including maternal age at delivery, race/ethnicity, pre‐pregnancy body mass index (BMI; kg/m^2^), history of a non‐live birth (previous stillbirth, termination, or other early pregnancy loss), multifetal pregnancy, pre‐existing hypertension, and pregestational diabetes. We used a change‐in estimate approach to minimize covariates and selected the most parsimonious multivariable model for stress and social support (Kleinbaum 2010), which controlled for maternal age and race/ethnicity.

Our main outcome of interest was stillbirth risk among all eligible birth defect cases combined (Table S2 includes the observed birth defects among those in the analysis). Given that the type of birth defect may modify the association between stress/social support and stillbirth risk, we stratified the results by birth defect when there were at least 3 exposed stillbirths with a given defect. Given the strong association of stillbirth with maternal race/ethnicity, we evaluated whether the associations between stress and social support measures and the risk for stillbirth differed by race/ethnicity on either the multiplicative or additive scales. To measure effect modification on the additive scale, we calculated the relative excess risk due to interaction (RERI) with 95% CIs based on standard errors calculated using the delta method (Knol and VanderWeele 2012). The RERI indicates presence and direction of effect modification, where 0 constitutes the null value. A value other than 0 indicates a departure from additivity but does not necessarily represent the magnitude of the effect modification. We assessed stress and race/ethnicity combinations using non‐Hispanic White women who reported low stress as the reference group. We assessed social support and race/ethnicity combinations using non‐Hispanic White women who reported high social support as the reference group. We assessed effect modification on the multiplicative scale by including product terms for maternal race/ethnicity and stress or social support in the adjusted models. We used a likelihood ratio test to determine the statistical significance of the maternal race/ethnicity interaction, comparing the model with the interaction terms to a model without them. Lastly, we performed a sensitivity analysis to evaluate potential bias due to the exclusion of elective terminations (Heinke et al. 2020). To do this, we repeated analyses after including all electively terminated pregnancies with a birth defect, first treating them as if they had been stillbirths and then treating them as if they had been live births. In this way, we produced a range of estimates and quantified the potential bias of excluding terminations under these two scenarios.

## 3 Results

There were 16,770 pregnancies with birth defects from NBDPS (2006–2011) and BD‐STEPS (2014–2021). After our exclusions, our final analytic sample included 148 stillbirths and 7417 live births (Figure 1). Among women experiencing pregnancies affected by stillbirth with birth defects, non‐Hispanic White women accounted for 42.6%, Hispanic women accounted for 38.5% and non‐Hispanic Black women accounted for 18.9% (Table 1). When compared to those with a live birth, women with a stillbirth more frequently reported non‐Hispanic Black or Hispanic race/ethnicity, education of < 12 years, and not having a previous pregnancy, but less frequently reported having a BMI ≥ 30, a previous live birth and alcohol use in the month before through the third month of pregnancy. Additionally, women with stillbirths more frequently completed the interview within 6 months of the estimated date of delivery than women with live births.

**TABLE 1:** Selected characteristics among stillbirths and livebirths with birth defects, National Birth Defects Prevention Study (2006–2011) and Birth Defects Study To Evaluate Pregnancy exposureS (2014–2021).

Maternal characteristics	Stillbirth (*n* = 148)	Livebirths (*n* = 7417)
	*n* (%)	*n* (%)
Race/ethnicity		
Non‐Hispanic White	63 (42.6)	4225 (57.0)
Non‐Hispanic Black	28 (18.9)	721 (9.7)
Hispanic	57 (38.5)	2471 (33.3)
Age at delivery		
< 20	20 (13.5)	740 (10.0)
20–24	38 (25.7)	1786 (24.1)
25–29	42 (28.4)	2009 (27.1)
30–34	31 (20.9)	1782 (24.0)
≥ 35	17 (11.5)	1100 (14.8)
Educational attainment		
< 12 years	36 (24.3)	1354 (18.3)
12 years	35 (23.6)	1913 (25.8)
> 12 years	76 (51.4)	4089 (55.1)
Pre‐pregnancy body mass index		
< 25.0	63 (42.6)	3600 (48.5)
25– < 30	50 (33.8)	1745 (23.5)
≥ 30	27 (18.2)	1647 (22.2)
# of previous pregnancies		
0	57 (38.5)	2297 (31.0)
1	41 (27.7)	1998 (26.9)
2	21 (14.2)	1382 (18.6)
≥ 3	29 (19.6)	1737 (23.4)
Previous livebirth	72 (48.6)	4223 (56.9)
Previous non‐live birth^a^	51 (34.5)	2572 (34.7)
Early pregnancy smoking^b^	25 (16.9)	1356 (18.3)
Early pregnancy alcohol use^b^	46 (31.1)	2966 (40.0)
Periconceptional vitamin use^c^	79 (53.4)	4268 (57.5)
Depression/anxiety medications	8 (5.4)	601 (8.1)
Any fertility treatment	7 (4.7)	430 (5.8)
Pre‐existing diabetes	5 (3.4)	197 (2.7)
Pre‐existing hypertension	28 (18.9)	1194 (16.1)
Study		
NBDPS	125 (84.5)	5135 (69.2)
BD‐STEPS	23 (15.5)	2282 (30.8)
Months from delivery to interview		
< 6	59 (39.9)	2293 (30.9)
7–12	34 (23.0)	2928 (39.5)
13–18	32 (21.6)	1482 (20.0)
≥ 19	18 (12.2)	604 (8.1)
Study center		
Arkansas	16 (10.8)	919 (12.4)
California	28 (18.9)	1090 (14.7)
Georgia	18 (12.2)	747 (10.1)
Iowa	11 (7.4)	696 (9.4)
Massachusetts	18 (12.2)	856 (11.5)
New York	5 (3.4)	756 (10.2)
North Carolina	22 (14.9)	988 (13.3)
Texas	12 (8.1)	519 (7.0)
Utah	18 (12.2)	846 (11.4)

Abbreviations: BD‐STEPS, Birth Defects Study To Evaluate Pregnancy exposureS; NBDPS, National Birth Defects Prevention Study.

^a^ Includes any other pregnancy that resulted in a non‐live birth (stillbirth, termination, miscarriage).

^b^ Early pregnancy is defined as the month before conception through the third month of pregnancy.

^c^ Periconceptional is defined as the month before conception through the first month of pregnancy.

Overall, women with stillbirths and women with live births had similar levels of high stress (16.9% vs. 18.2%) and low social support (34.5% vs. 28.3%) (Table 2). Regardless of stillbirth or livebirth status, the most commonly reported stressor was stress due to serious relationship difficulties with a partner, and the least commonly reported social support was financial support. High stress was not significantly associated with an increased risk of stillbirth (adjusted RR 0.83, 95% CI 0.54–1.28), nor was low social support (adjusted RR 1.17, 95% CI 0.83–1.67). The individual stressors and individual social supports were also not associated with stillbirth in the adjusted analysis (Table 2). Analyses examining the association of stress and social support in combination did not find significant associations with stillbirth.

**TABLE 2:** The effect of stress and social support on stillbirth with birth defects, National Birth Defects Prevention Study (2006–2011) and Birth Defects Study To Evaluate Pregnancy exposureS (2014–2021).

	Stillbirth (*n* = 148)	Live birth (*n* = 7417)	Crude RR (95% CI)	Adjusted RR^a^ (95% CI)
	*n* (%)	*n* (%)		
High stress^b^	25 (16.9)	1352 (18.2)	0.91 (0.60, 1.40)	0.83 (0.54, 1.28)
Stress due to a death of a close friend/family	15 (10.1)	1080 (14.6)	0.67 (0.39, 1.13)	0.63 (0.37, 1.07)
Stress due to illness or injury of close friend, family, self	12 (8.1)	957 (12.9)	0.60 (0.33, 1.08)	0.59 (0.33, 1.07)
Stress due to serious legal or financial problems	26 (17.6)	1131 (15.3)	1.18 (0.78, 1.79)	1.12 (0.74, 1.70)
Stress due to serious relationship difficulties with partner	38 (25.7)	1415 (19.1)	1.45 (1.01, 2.09)	1.27 (0.87, 1.84)
Stress due to being or knowing a victim of abuse, violence, or crime	8 (5.4)	505 (6.8)	0.79 (0.39, 1.59)	0.70 (0.34, 1.42)
Low social support^c^	51 (34.5)	2095 (28.3)	1.33 (0.95, 1.86)	1.17 (0.83, 1.67)
Lack of financial support	36 (24.3)	1317 (17.8)	1.48 (1.02, 2.14)	1.32 (0.90, 1.94)
Lack of emotional support	27 (18.2)	1264 (17.0)	1.08 (0.72, 1.64)	0.93 (0.61, 1.43)
Lack of support with daily tasks	29 (19.6)	1283 (17.3)	1.16 (0.78, 1.74)	1.05 (0.70, 1.59)
Stress * Social support				
Low stress, high support	87 (56.8)	4554 (61.4)	Reference	Reference
Low stress, low support	39 (26.4)	1511 (20.4)	1.39 (0.95, 2.02)	1.23 (0.83, 1.82)
High stress, high support	13 (8.8)	768 (10.4)	0.92 (0.52, 1.64)	0.84 (0.47, 1.51)
High stress, low support	12 (8.1)	584 (7.9)	1.11 (0.61, 2.02)	0.93 (0.51, 1.71)

Abbreviations: CI, confidence interval; RR, relative risk.

^a^ Adjusted for maternal age at delivery and race/ethnicity.

^b^ High stress was defined as reporting 3–5 stressful life events.

^c^ Low social support was defined as reporting 0–2 social supports.

Neither the effect of high stress nor low social support on stillbirth risk statistically differed by type of birth defect, although numbers were small (Table 3). While the crude RR for stillbirth associated with high stress ranged from 0.32 for pregnancies affected by gastroschisis to 1.73 for pregnancies affected by omphalocele, all 95% CIs included the null. Similarly, the crude RR for stillbirth associated with low social support ranged from 1.09 for pregnancies affected by gastroschisis to 2.86 for pregnancies affected by congenital heart defects.

**TABLE 3:** The effect of stress and social support on stillbirth with birth defects, stratified by birth defect, National Birth Defects Prevention Study (2006–2011) and Birth Defects Study To Evaluate Pregnancy exposureS (2014–2021).^a^

	Stillbirth	Live birth	Crude RR (95% CI)
	Exposed/unexposed	Exposed/unexposed	
High stress^b^			
Amniotic band sequence	4/11	23/63	1.00 (0.29, 3.44)
Spina bifida	3/13	121/572	1.09 (0.31, 3.89)
Cleft lip or palate	4/23	427/2215	0.90 (0.31, 2.62)
Gastroschisis	3/26	225/627	0.32 (0.10, 1.07)
Omphalocele	3/5	33/95	1.73 (0.39, 7.63)
Congenital heart defects	3/8	142/576	1.54 (0.40, 5.81)
Low social support^c^			
Spina bifida	6/10	211/482	1.37 (0.49, 3.82)
Cleft lip or palate	10/17	702/1940	1.63 (0.74, 3.57)
Limb deficiency	5/9	154/376	1.36 (0.45, 4.11)
Gastroschisis	9/20	249/603	1.09 (0.49, 2.43)
Omphalocele	3/5	38/90	1.42 (0.32, 6.25)
Congenital heart defects	6/5	212/506	2.86 (0.86, 9.49)

Abbreviations: CI, confidence interval; RR, relative risk.

^a^ Sample size (*n* < 3) prohibited analysis of the combined stress and social support exposure in this stratified analysis.

^b^ High stress was defined as reporting 3–5 stressful life events.

^c^ Low social support was defined as reporting 0–2 social supports.

The RR estimate for the association between stress and the risk of stillbirth did not differ by maternal race/ethnicity on either the additive or multiplicative scales (Table 4). In the analysis of additive interaction, the 95% CIs within each stratum of stress and race/ethnicity were wide and overlapped between groups. Compared to non‐Hispanic White women with low/no stress, the risk of stillbirth was significantly increased among non‐Hispanic Black women, regardless of their reported stress (adjusted RR for high stress: 2.76, 95% CI 1.38, 5.19 and adjusted RR for low/no stress: 2.13, 95% CI 1.28, 3.58) (Table 4). Hispanic women, regardless of their reported stress, did not have a higher risk of stillbirth compared to non‐Hispanic White women. Additionally, the CIs for the RR estimates for stress and stillbirth within race/ethnicity strata all included the null; multiplicative interaction tests were not statistically significant (likelihood ratio test *p*‐value = 0.3988).

**TABLE 4:** The joint effect of high stress and race/ethnicity on risk of stillbirth with birth defects, National Birth Defects Prevention Study (2006–2011) and Birth Defects Study To Evaluate Pregnancy exposureS (2014–2021).

	Low stress		High stress		RR (95% CI) for stress, within race/ethnicity strata
	Stillbirth/live birth	RR (95% CI)	Stillbirth/live birth	RR (95% CI)	
Non‐Hispanic Black	19/531	2.13 (1.28, 3.58)	9/190	2.76 (1.38, 5.19)	1.29 (0.60, 2.81)
Hispanic	48/2002	1.43 (0.97, 2.11)	9/469	1.13 (0.56, 2.29)	0.79 (0.39, 1.60)
Non‐Hispanic White	56/3532	Reference	7/693	0.61 (0.28, 1.33)	0.61 (0.28, 1.33)

*Note:* Measure of additive effect modification: RERI non‐Hispanic Black versus non‐Hispanic White = 1.02 (−1.03, 3.07); RERI Hispanic versus non‐Hispanic White = 0.09 (−0.89, 1.07). Measure of multiplicative effect modification: Likelihood ratio test *p*‐value = 0.3988. *p*‐value for product term: non‐Hispanic Black versus non‐Hispanic White = 0.1816; Hispanic versus non‐Hispanic White = 0.6287. RRs are adjusted for maternal age.

Abbreviations: CI, confidence interval; RR, relative risk.

Similarly, the RR estimates for the association between social support and the risk for stillbirth did not differ by race/ethnicity on either the multiplicative or additive scale. The 95% CIs within each stratum of social support and race/ethnicity were wide and overlapped between groups. Compared to non‐Hispanic White women with high social support, the risk of stillbirth was significantly increased among non‐Hispanic Black women who reported high social support (adjusted RR 2.36, 95% CI 1.36, 4.08), and the RR was further from the null among non‐Hispanic Black women who reported low social support (adjusted RR 3.25, 95% CI 1.71, 6.18) (Table 5). Hispanic women who reported high social support (adjusted RR 1.61, 95% CI 1.03, 2.53), and those who reported low social support (adjusted RR 1.55, 95% CI 0.96, 2.48) had similar elevations in the risk of stillbirth. The CIs for the RR estimates for social support and stillbirth within race/ethnicity strata all included the null; multiplicative interaction tests were not statistically significant (likelihood ratio test *p*‐value = 0.5754).

**TABLE 5:** The joint effect of low social support and race/ethnicity on risk of stillbirth with birth defects, National Birth Defects Prevention Study (2006–2011) and Birth Defects Study To Evaluate Pregnancy exposureS (2014–2021).

	High support		Low support		RR (95% CI) for support, within race/ethnicity strata
	Stillbirth/live birth	RR (95% CI)	Stillbirth/live birth	RR (95% CI)	
Non‐Hispanic Black	17/491	2.36 (1.36, 4.08)	11/230	3.25 (1.71, 6.18)	1.38 (0.65, 2.89)
Hispanic	31/1307	1.61 (1.03, 2.53)	26/1164	1.55 (0.96, 2.48)	0.96 (0.57, 1.60)
Non‐Hispanic White	49/3524	Reference	14/701	1.40 (0.77, 2.52)	1.40 (0.77, 2.52)

*Note:* Measure of additive effect modification: RERI non‐Hispanic Black versus non‐Hispanic White = 0.49 (−1.81, 2.79); RERI Hispanic versus non‐Hispanic White = −0.47 (−1.63, 0.70). Measure of multiplicative effect modification: Likelihood ratio test *p*‐value = 0.5754. *p*‐value for product term: non‐Hispanic Black versus non‐Hispanic White = 0.9727; Hispanic versus non‐Hispanic White = 0.3426. RRs are adjusted for maternal age.

Abbreviations: CI, confidence interval; RR, relative risk.

We examined risks of stillbirth for the combination of stress and social support by race/ethnicity (Table S3). As with other additive interaction analyses, the 95% CIs overlapped between groups. None of the multiplicative or additive interaction tests were statistically significant. Among non‐Hispanic Black women, we observed elevated risk of stillbirth regardless of the combination of stress and social support. The highest risk of stillbirth was among non‐Hispanic Black women with high stress and low support (adjusted RR 3.48, 95% CI 1.40–8.61), although risk was also significantly elevated among those with low stress and low support (adjusted RR 2.73, 95% CI 1.18–6.30) and low stress and high support (adjusted RR 2.13, 95% CI 1.15–3.93).

Lastly, we performed a sensitivity analysis to address potential bias due to the exclusion of 175 elective terminations from the main analysis. The inclusion of these terminations as stillbirths and then as live births did not meaningfully alter the RR estimates of high stress or low social support (Table S4).

## 4 Discussion

We examined the association between stress, social support, and stillbirth among pregnancies affected by birth defects, and examined if the RR differed by type of birth defect or maternal race/ethnicity. We found that neither high stress nor low social support was associated with an increased risk of stillbirth among those with birth defects. We found that the RR between these measures and stillbirth risk did not vary by type of birth defect. Additionally, we observed that our RR estimates did not differ by maternal race/ethnicity. We observed that non‐Hispanic Black women had a higher risk of stillbirth regardless of their stress or social support levels. We did not observe that high social support impacted the risk from high stress overall or among any racial/ethnic group.

In the general obstetric population, the Stillbirth Collaborative Research Network, a population‐based case–control study of women with a stillbirth, observed that women who reported experiencing high levels of self‐reported stress in pregnancy had over two times the odds of a stillbirth compared to those who reported no stress (Hogue et al. 2013). Similarly, a cohort study in Denmark observed that women with a high level of stress had an 80% increased risk of stillbirth compared with women with a lower level of stress during pregnancy (Wisborg et al. 2008). We did not observe an overall increased risk of stillbirth among pregnancies with birth defects related to stress and/or social support; however, among non‐Hispanic Black women with pregnancies with birth defects, the RR for those with high stress and those with low social support was farthest from the null. Although the direct relationship between social support and stillbirth has not been thoroughly explored in the general obstetric population, multiple studies have observed that greater emotional support is associated with lower levels of perceived stress, anxiety, and depression during pregnancy (Wang et al. 2023; Al‐Mutawtah et al. 2023), suggesting that social support could mitigate the harmful effects of stress (Hogue et al. 2013; Hogue 2016; East et al. 2019). However, in our study, high social support did not appear to impact the magnitude of the RR of stillbirth associated with high stress.

There were some limitations in our study. Both studies collected data on stress and social support experienced during the 6 months surrounding conception. While interviews were conducted up to 2 years after delivery, the majority of interviews were conducted within a year of delivery (63% of stillbirths and 71% of live births). Some misclassification may have occurred if women did not accurately recall these exposures, and if maternal recall differed by birth outcome, recall bias may have influenced our findings. Yet, we suspect the impact is minimal as the questions focused on major life events which likely reduces susceptibility to recall error and the prevalence of reported stress/social supports within our study is similar to levels observed in other US pregnant populations (Hogue et al. 2013; Burns et al. 2015). Our analysis was limited by small numbers, which impacted the analysis in several ways. While the magnitude of the RR among non‐Hispanic Black women with high stress was further from the null than the RR among non‐Hispanic Black women with low stress, the interaction results were not statistically significant on either scale. Effect measure modification assessments are often poorly powered especially for rare outcomes, making it difficult to detect true effects (Marshall 2007; VanderWeele and Knol 2014). Additionally, the effects of stressful life events and aspects of social support are thought to be cumulative (Carmichael et al. 2017; McLean et al. 1993); due to small numbers we examined stress and social support as dichotomous instead of ordinal variables. The sample size also limited our ability to fully explore potential confounding.

Although we did not observe strong confounding by measured factors, it remains possible that estimates are biased in either direction by unmeasured confounders, which limits the generalizability of our findings. Additionally, our focus on stillbirths with birth defects may have caused bias resulting from collider stratification (Banack and Kaufman 2014). Yet, such bias could only exist if there was an association between stress and birth defects within NBDPS. The association between high stress and the birth defects included in our analysis (Table S2) is not clear, as previous NBDPS analyses have not found consistent statistically significant associations with birth defects (Carmichael et al. 2014). Nevertheless, our analysis may be biased due to its focus on stillbirth with birth defects and our results should not be generalized to all pregnancies. Lastly, we excluded women who were missing race/ethnicity or reported a race/ethnicity other than non‐Hispanic White, non‐Hispanic Black, or Hispanic, which reduces the generalizability of our results.

A key strength of this study is the use of data from two large, population‐based case–control studies to explore the risk of stillbirth among those with birth defects. We were able to evaluate the associations across different races/ethnicities. Additionally, we were able to conduct analyses exploring stress and social support in combinations and quantifying the impacts of the exclusion of pregnancy terminations on our results. Our study focuses on stillbirths among pregnancies with a birth defect diagnosis, a population not often studied. In addition to excluding birth defects that are infrequently diagnosed or are otherwise unlikely to be diagnosed consistently in stillbirths (Heinke et al. 2020), as well as anencephaly, we explored whether the RR between our stress and social support measures and stillbirth differed by specific birth defect. We found that there was no evidence of variation in the risk estimate by birth defect.

Overall, we found that stress and social support were not associated with stillbirth in fetuses with birth defects. While the RR was largest in magnitude among non‐Hispanic Black women who experience the highest levels of stress and lowest levels of social support, we did not observe statistically significant effect modification by race/ethnicity.

## Author Contributions

M.M.H.: conceptualization, methodology, formal analysis, writing – original draft, visualization, supervision, funding acquisition. J.M.S.: validation; writing – review and editing. E.M.W.: formal analysis; validation; writing – review and editing. U.M.R., E.A.P., S.L.C., C.M.C., A.J.A., N.E., W.N.N., S.C.F.: writing – review and editing.

## Funding

This project was supported through Centers for Disease Control and Prevention (CDC) cooperative agreements under PA #96043, PA #02081, FOA #DD09‐001, FOA #DD13‐003, NOFO #DD18‐001, and NOFO #DD23‐001 to the Centers for Birth Defects Research and Prevention participating in the National Birth Defects Prevention Study and/or the Birth Defects Study To Evaluate Pregnancy exposureS and to the New York Center for Birth Defects Research and Prevention U01 DD001227, U01 DD001304.

## Ethics Statement

All interviewed study participants provided informed consent. The Centers for Disease Control and Prevention Institutional Review Board (IRB), along with the IRBs for each participating site, has approved the NBDPS. The Centers for Disease Control and Prevention IRB has approved the BD‐STEPS.

## Conflicts of Interest

The authors declare no conflicts of interest.

## Supporting information


**Table S1:** Text of questions related to stress social support in the National Birth Defects Prevention Study (2006–2011) and Birth Defects Study To Evaluate Pregnancy exposureS (2014–2021).
**Table S2:** Distribution of stillbirths and livebirths by birth defect, National Birth Defects Prevention Study (2006–2011) and Birth Defects Study To Evaluate Pregnancy exposureS (2014–2021).
**Table S3:** The joint effect of stress/social support and race/ethnicity on risk of stillbirth with birth defects, National Birth Defects Prevention Study (2006–2011) and Birth Defects Study To Evaluate Pregnancy exposureS (2014–2021).
**Table S4:** The association between stress and social support on stillbirth with birth defects when including terminations (*n* = 175) as stillbirths and terminations as livebirths, National Birth Defects Prevention Study (2006–2011) and Birth Defects Study To Evaluate Pregnancy exposureS (2014–2021).

## Data Availability

The study questionnaires and process for accessing the data used in this study are described at https://www.cdc.gov/birth‐defects/php/bd‐steps‐nbdps‐data/index.html. The code book and analytic code may be made available upon request.
