# Supplementary material for: Maternal Stressors and Social Support in Early Pregnancy and the Risk of Stillbirth Among Fetuses With Birth Defects: Exploring Effect Modification by Race/Ethnicity
Source: Birth Defects Res. 2026 Jul 25;118(8):e70096. doi: 10.1002/bdr2.70096 (PMC13401088; doi:10.1002/bdr2.70096)
Supplement: Supplementary file 1 — Table S1: Text of questions related to stress social support in the National Birth Defects Prevention Study (2006–2011) and Birth Defects Study To Evaluate Pregnancy exposureS (2014–2021). Table S2: Distribution of stillbirths and livebirths by birth defect, National Birth Defects Prevention Study (2006–2011) and Birth Defects Study To Evaluate Pregnancy exposureS (2014–2021). Table S3: The joint effect of stress/social support and race/ethnicity on risk of stillbirth with birth defects, National Birth Defects Prevention Study (2006–2011) and Birth Defects Study To Evaluate Pregnancy exposureS (2014–2021). Table S4: The association between stress and social support on stillbirth with birth defects when including terminations (n = 175) as stillbirths and terminations as livebirths, National Birth Defects Prevention Study (2006–2011) and Birth Defects Study To Evaluate Pregnancy exposureS (2014–2021). [file BDR2-118-e70096-s001.docx]

**Supplemental Table 1. Text of questions related to stress social support in the** **National Birth Defects Prevention Study (2006-2011) and Birth Defects Study To Evaluate Pregnancy exposureS (2014-2021).**

From three months before you became pregnant through your third month of pregnancy:

| *Stress* |
| --- |
| Did you experience any serious relationship difficulties with your husband or partner or become  separated or divorced? |
| Did you or your husband or partner have any serious legal or financial problems? |
| Were you or someone close to you a victim of abuse, violence, or crime? |
| Did you or someone close to you have a serious illness or injury? |
| Did someone close to you die? |
| *Social Support* |
| Could you count on anyone to provide you with emotional support such as talking over a  problem or helping with a difficult decision, if you had needed it? |
| Could you count on anyone to provide you with help financially such as paying bills or providing  food or clothes, if you had needed it? |
| Could you count on anyone to provide you with help with daily tasks such as grocery shopping,  childcare, or cooking, if you had needed it? |

**Supplemental Table 2.** **Distribution of stillbirths and livebirths by birth defect, National Birth Defects Prevention Study (2006-2011) and Birth Defects Study To Evaluate Pregnancy exposureS (2014-2021).^a^**

| **Birth Defect** | **Stillbirth**  **(n=148)** | | **Live birth (n=7,417)_** | |
| --- | --- | --- | --- | --- |
|  | **n** | **%** | **n** | **%** |
| Renal agenesis | 9 | 16.7 | 45 | 83.3 |
| Amniotic band sequence | 15 | 14.9 | 86 | 85.1 |
| Omphalocele | 11 | 7.9 | 128 | 92.1 |
| Double outlet right ventricular defect |  | 7.7 |  | 92.3 |
| Cloacal exstrophy |  | 6.9 |  | 93.1 |
| Single ventricle defects |  | 5.9 |  | 94.1 |
| Atrioventricular septal defect |  | 4.8 |  | 95.2 |
| Encephalocele | 3 | 4.1 | 71 | 95.9 |
| Holoprosencephaly | 3 | 4.1 | 71 | 95.9 |
| Transverse limb deficiency | 13 | 3.7 | 343 | 96.3 |
| Heterotaxy | 4 | 3.6 | 108 | 96.4 |
| Gastroschisis | 29 | 3.3 | 852 | 96.7 |
| Diaphragmatic hernia | 14 | 2.9 | 476 | 97.1 |
| Spina bifida | 16 | 2.3 | 693 | 97.7 |
| Hypoplastic heart syndrome |  | 2.1 |  | 97.9 |
| Anophthalmos/microphthalmos |  | 1.5 |  | 98.5 |
| Dandy-Walker malformation |  | 1.4 |  | 98.6 |
| Cleft lip with or without palate |  | 1.1 |  | 98.9 |
| Hydrocephaly | 19 | 1.1 | 1748 | 98.9 |
| Coarctation of the aorta | 2 | 1.1 | 188 | 98.9 |
| Cleft palate only | 27 | 0.9 | 2642 | 99.1 |
| Duodenal atresia |  | 0.9 |  | 99.1 |
| Tetralogy of Fallot |  | 0.9 |  | 99.1 |
| Longitudinal limb deficiency | 8 | 0.6 | 894 | 99.4 |
| Esophageal atresia |  | 0.4 |  | 99.6 |
| Anotia/microtia |  | 0.2 |  | 99.8 |
| ^a^We have suppressed the counts of stillbirths and livebirths with each type of birth defect if any of the cell sizes are < 3. In these instances, we have only presented the % of each birth defect that is stillbirth and live birth | | | | |

**Supplemental Table 3. The joint effect of stress/social support and race/ethnicity on risk of stillbirth with birth defects, National Birth Defects Prevention Study (2006-2011) and Birth Defects Study To Evaluate Pregnancy exposureS (2014-2021).**

|  | **Low stress, high support** | | **Low stress, low support** | | **High stress, high support** | | **High stress, low support** | |
| --- | --- | --- | --- | --- | --- | --- | --- | --- |
|  | Stillbirth/  live birth | RR (95% CI) | Stillbirth/  live birth | RR (95% CI) | Stillbirth/  live birth | RR (95% CI) | Stillbirth/  live birth | RR (95% CI) |
| Non-Hispanic Black | 13/389 | 2.13 (1.15, 3.93) | 6/142 | 2.73 (1.18, 6.30) | 4/102 | 2.48 (0.91, 6.79) | 5/88 | 3.48 (1.40, 8.61) |
| Hispanic | 26/1121 | 1.50 (0.90, 2.48) | 22/881 | 1.48 (0.83, 2.66) | 5/186 | 1.45 (0.52, 4.04) | 4/283 | 0.98 (0.35, 2.73) |
| Non-Hispanic White | 45/3044 | Reference | 11/488 | 1.47 (0.77, 2.83) | 4/480 | 0.53 (0.19, 1.48) | 3/213 | 0.91 (0.28, 2.91) |
| RR = relative risk; CI = confidence interval.  Measures of additive effect modification, RERI (95% CI): non-Hispanic Black vs. non-Hispanic White for high stress, low support: 1.44 (-1.90, 4.78);  Hispanic vs. non-Hispanic White for high stress, low support: -0.40 (-1.96, 1.15); non-Hispanic Black vs. non-Hispanic White for high stress, high support: 0.76 (-2.05, 3.58); Hispanic vs. non-Hispanic White for high stress, high support: 0.64 (-0.94, 2.23); non-Hispanic Black vs. non-Hispanic White for low stress, low support: 0.12 (-2.77, 3.00); Hispanic vs. non-Hispanic White for low stress, low support: -0.62 (-2.04, 0.78).  Measures of multiplicative effect modification: Likelihood ratio test p-value = 0. 68810.  P--value for product term: non-Hispanic Black vs. non-Hispanic White for high stress, low support: 0.4547 Hispanic vs. non-Hispanic White for high stress, low support: 0.6233; non-Hispanic Black vs. non-Hispanic White for high stress, high support: 0.3087; Hispanic vs. non-Hispanic White for high stress, high support: 0.2961; non-Hispanic Black vs. non-Hispanic White for low stress, low support: 0.8133; Hispanic vs. non-Hispanic White for low stress, low support: 0.4900. | | | | | | | | |

**Supplemental Table 4. The association between stress and social support on stillbirth with birth defects when including terminations (n=175) as stillbirths and terminations as livebirths, National Birth Defects Prevention Study (2006-2011) and Birth Defects Study To Evaluate Pregnancy exposureS (2014-2021)..**

|  | **Including terminations as stillbirths** | | **Including terminations as livebirths** | |  |
| --- | --- | --- | --- | --- | --- |
|  | **Stillbirth/**  **live birth**  **(323/7,417)** | **Adjusted RR**^a^  **(95% CI)** | **Stillbirth/**  **live birth**  **(148/7,592)** | **Adjusted RR**^a^  **(95% CI)** |  |
| **High stress** | 60/1352 | 1.01 (0.77, 1.33) | 25/1387 | 0.83 (0.54, 1.27) |  |
| Stress due to a death of a close friend/family | 38/1080 | 0.78 (0.56, 1.09) | 15/1103 | 0.63 (0.37, 1.07) |  |
| Stress due to illness or injury of close friend, family, self | 36/957 | 0.84 (0.60, 1.18) | 12/981 | 0.59 (0.33, 1.07) |  |
| Stress due to serious legal or financial problems | 49/1131 | 0.99 (0.73, 1.33) | 26/1154 | 1.12 (0.74, 1.70) |  |
| Stress due to serious relationship difficulties with partner | 75/1415 | 1.27 (0.98, 1.64) | 38/1452 | 1.26 (0.87, 1.83) |  |
| Stress due to being or knowing a victim of abuse, violence, or crime | 23/505 | 1.04 (0.69, 1.58) | 8/520 | 0.69 (0.34, 1.41) |  |
| **Low social support** | 88/2095 | 0.95 (0.74, 1.23) | 51/2132 | 1.18 (0.83, 1.67) |  |
| Lack of financial support | 57/1317 | 1.00 (0.75, 1.33) | 36/1338 | 1.33 (0.91, 1.95) |  |
| Lack of emotional support | 46/1264 | 0.81 (0.59, 1.11) | 27/1283 | 0.94 (0.61, 1.44) |  |
| Lack of support with daily tasks | 57/1283 | 1.03 (0.77, 1.38) | 29/1311 | 1.05 (0.70, 1.59) |  |
| **Stress*Social support** |  |  |  |  |  |
| Low stress, high support | 207/4554 | Reference | 84/4672 | Reference |  |
| Low stress, low support | 61/1511 | 0.92 (0.68, 1.23) | 39/1533 | 1.24 (0.84, 1.84) |  |
| High stress, high support | 33/768 | 0.96 (0.67, 1.38) | 13/788 | 0.85 (0.47, 1.50) |  |
| High stress, low support | 27/584 | 1.03 (0.69, 1.53) | 12/599 | 0.93 (0.51, 1.71) |  |
| RR = relative risk; CI = confidence interval  ^a^ Adjusted for maternal age at delivery and race/ethnicity. | | | | | |
